# Challenging the principle of utility as a barrier for wider use of liver transplantation for hepatocellular cancer

**DOI:** 10.1245/s10434-017-5989-x

**Published:** 2017-07-10

**Authors:** Michał Grąt, Jan Stypułkowski, Waldemar Patkowski, Karolina M. Wronka, Emil Bik, Maciej Krasnodębski, Łukasz Masior, Zbigniew Lewandowski, Michał Wasilewicz, Karolina Grąt, Marek Krawczyk, Krzysztof Zieniewicz

**Affiliations:** 1 Department of General, Transplant and Liver Surgery, Medical University of Warsaw, Warsaw, Poland; 2 Department of Epidemiology, Medical University of Warsaw, Warsaw, Poland; 3 Hepatology and Internal Medicine Unit, Department of General, Transplant and Liver Surgery, Medical University of Warsaw, Warsaw, Poland; 4 Second Department of Clinical Radiology, Medical University of Warsaw, Warsaw, Poland

## Abstract

**Background:**

Although transplant benefit appears superior for patients with advanced hepatocellular cancer (HCC), liver transplantation remains limited to selected low-risk HCC patients to keep their outcomes similar to heterogeneous group of non-HCC patients. The purpose of this study was to assess the rationale for current policy of restricting access to liver transplantation to minority of HCC patients based on utility principle.

**Methods:**

This retrospective cohort study comprised 1246 liver transplant recipients, including 206 HCC and 1040 non-HCC patients. Patient survival was the primary outcome measure. Patients with HCC and benign diseases were divided into low-, moderate-, and high-risk subgroups basing on independent risk factors for disease-free survival and model for end-stage liver disease (MELD) score (<30, 30–40, >40), respectively.

**Results:**

MELD (*p* < 0.001) and presence of HCC (*p* = 0.008) were independent risk factors for early and late mortality, respectively. Total tumor volume (*p* = 0.008) and alpha-fetoprotein (*p* = 0.013) were independent predictors of recurrence and mortality used for division of HCC patients into low-, moderate-, and high-risk subgroups, with disease-free survival rates of 74.9% (5 years), 51.7% (5 years), and 8.0% (3 years), respectively (*p* < 0.001). There were no differences in 5-year overall survival between low-risk HCC (74.9%) and non-HCC (81.9%) patients (*p* = 0.210), moderate-risk HCC (63.3%) and non-HCC (68.0%) patients (*p* = 0.372), and high-risk HCC (55.0%) and non-HCC (56.0%) patients (*p* = 0.559).

**Conclusions:**

The principle of utility is unequally applied for restriction of access to liver transplantation for HCC patients. The results provide rationale for discussion on reinitiation of liver transplantation for advanced HCCs.

**Electronic supplementary material:**

The online version of this article (doi:10.1245/s10434-017-5989-x) contains supplementary material, which is available to authorized users.

Liver transplantation (LT) is an effective treatment of patients with various diseases, including hepatocellular cancer (HCC).^1^ However, scarcity of donors enforced application of stringent selection and allocation processes, which should be based on urgency and utility.^2^,^3^ Eligibility of HCC patients for LT based on the Milan criteria form a direct application of the utility principle in the selection process.^4^ Although numerous studies indicate that the criteria may be safely expanded, this treatment remains reserved for a minority of HCC patients.^5^–^13^ Further expansion of eligibility criteria is controversial due to the expected unacceptable posttransplant outcomes of patients with advanced tumors, not supporting its most probable negative impact on waiting time and pretransplant mortality of patients with benign indications.^14^ In fact, the position of HCC patients who fulfill the current selection criteria is characterized by lower pretransplant mortality and higher likelihood of receiving LT.^15^,^16^

Aside from the distinct considerations of urgency and utility, the transplant benefit for HCC patients currently appears lower than for those with benign indications.^17^ However, patients at higher Barcelona Clinic Liver Cancer stages experience increased benefits from transplantation.^18^ Therefore, more advanced HCCs may paradoxically be a better indication for LT than tumors within the Milan criteria. To evaluate the future perspective of potential liberalization of selection criteria according to the utility principle, the purpose of this study was to compare survival outcomes after LT between HCC and non-HCC patients with respect to the risk profile of both populations.

## Materials and Methods

A total of 1387 LTs were performed in the Department of General, Transplant and Liver Surgery (Medical University of Warsaw) between January 2001 and July 2014. Following exclusion of retransplantations (*n* = 87) and LTs in patients with non-HCC tumors (*n* = 54), this retrospective cohort study was based on 1246 LTs. Of these, 206 and 1040 were performed in HCC and non-HCC patients, respectively. The study was approved by the local ethics committee.

Patient survival was the primary outcome measure that was assessed at fifth posttransplant year. Disease-free survival was a secondary outcome measure for HCC patients applied for estimating their risk profile. It was calculated from transplantation until HCC recurrence or patient death (combined endpoint). For the purposes of all analyses, observations were censored at the last available follow-up or at 5 years after transplantation (whichever occurred first). Information on the operative technique, immunosuppression protocol, and posttransplant follow-up was provided previously.^19^–^21^

Risk factors for inferior posttransplant survival were first examined in all patients, with special reference to Model for End-stage Liver Disease (MELD) score and presence of HCC. Separate analyses were performed to evaluate factors associated with early (90-day) and late (observation beginning at 91th posttransplant day) mortality. In HCC patients, risk factors for worse 5-year disease-free survival were established. Based on the results of these analyses, both HCC and non-HCC patient populations were divided into low-risk, moderate-risk, and high-risk subgroups. Division of HCC patients was performed using the established cutoffs for independent predictors. Division of non-HCC patients was performed based on MELD in an exploratory fashion to search for subgroups with survival outcomes similar to HCC patients. For the purposes of outcome analyses, HCC patients were additionally divided into subgroups with 0–2, 3–4, and >4 points according to the AFP model.^6^

Continuous variables were presented as medians (interquartile ranges) and categorical variables were presented as frequencies. Mann–Whitney *U* test and χ^2^ test were used for intergroup comparisons, as appropriate. Survival outcomes were estimated with the Kaplan–Meier method and compared with log-rank test. Risk factors for early mortality were assessed with logistic regression. Risk factors for late mortality and inferior disease-free survival were evaluated with Cox proportional hazards regression. Optimal cutoffs for continuous variables in prediction of HCC recurrence were based on receiver operating characteristics (ROC) analyses. Odds ratios (ORs), hazard ratios (HRs), and areas under the curves (AUCs) were presented with 95% confidence intervals (95% confidence interval [CI]). The level of significance was set at 0.05. Analyses were computed with STATISTICA v. 12 (StatSoft Inc, Tulsa, USA) and SAS v. 9.4 (SAS Institute, Cary, NC).

## Results

Baseline characteristics of patients included in the study are presented in Table 1. Compared with non-HCC patients, HCC patients were characterized by older age, increased frequency of males, lower MELD score, increased rate of hepatitis C virus and hepatitis B virus infections, near zero rate of cholestatic diseases, transplantations performed with higher transplant team experience, less intraoperative transfusions, and older donor age (all *p* < 0.001). With the median follow-up of 47.2 months, 228 patients died over the 5 posttransplant years. Postoperative (90-day) mortality was 8.4% (105/1246). Patient survival at 5 years for HCC and non-HCC recipients was 65.2 and 78.5%, respectively (*p* = 0.044, Supplementary Fig. 1).

**Table 1:** Comparisons of baseline characteristics between hepatocellular cancer patients and those with benign indications included in the study cohort

Factors	HCC patients (*n* = 206)	Non-HCC patients (*n* = 1040)	*P*
Recipient sex			<0.001
Male	147 (71.4%)	545 (52.4%)	
Female	59 (28.6%)	495 (47.6%)	
Recipient age (yr)	57 (52–61)	46 (34–54)	<0.001
MELD score	11 (8–13)	14 (10–21)	<0.001
Hepatitis C virus infection	142 (68.9%)	247 (23.8%)	<0.001
Hepatitis B virus infection	83 (40.3%)	185 (17.8%)	<0.001
Alcoholic liver disease	34 (16.6%)	214 (20.6%)	0.191
Primary sclerosing cholangitis	0 (0.0%)	131 (12.6%)	<0.001
Primary biliary cirrhosis	1 (0.5%)	101 (9.7%)	<0.001
Experience of the transplant team^a^	957 (656–1235)	709 (390–1076)	<0.001
Intraoperative PRBC transfusions (units)	3 (2–6)	4 (2–8)	<0.001
Intraoperative FFP transfusions (units)	7 (5–10)	8 (6–10)	<0.001
Duration of cold ischemia (hr)	9.0 (8.0–10.4)	9.0 (7.8–10.2)	0.193
Donor age (yr)	49 (37–57)	44 (31–53)	<0.001
Within the Milan criteria	122 (59.2%)		
Within the up-to-7 criteria	157 (76.2%)		
Within the UCSF criteria	147 (71.4%)		
Size of the largest tumor (mm)	30 (20–45)		
Number of tumors	1 (1–3)		
Total tumor volume (cm^3^)	22.5 (5.2–54.7)		
Microvascular invasion	57 (27.7%)		
Poor tumor differentiation	24 (11.7%)		
Alpha-fetoprotein (ng/ml)	15.7 (6.1–144.4)		
Neoadjuvant treatment	87 (42.2%)		

Data are presented as *n* (%) or median (interquartile range)

^a^Defined as a cumulative number of previously performed transplantations in the department

*HCC* hepatocellular cancer, *MELD* model for end-stage liver disease, *PRBC* packed red blood cells, *FFP* fresh frozen plasma, *UCSF* University of California, San Francisco

In all patients, MELD score (*p* < 0.001), increased intraoperative blood transfusions (*p* = 0.022), and longer duration of cold ischemia (*p* = 0.013) were independent predictors of early mortality (Table 2). Presence of HCC (*p* = 0.008) and increased intraoperative blood transfusions (*p* = 0.007) were independent predictors of late mortality.

**Table 2:** Analyses of risk factors for worse patient survival after liver transplantation in the entire study cohort

Factor	90-day mortality		Long-term survival: >90 days–5 years	
	OR (95% CI)	*P*	HR (95% CI)	*P*
Univariable analyses				
Hepatocellular cancer	0.83 (0.47–1.46)	0.518	2.11 (1.40–3.18)	<0.001
MELD score	2.29 (1.89–2.78)	<0.001	1.01 (0.80–1.27)	0.934
Male recipient sex	0.62 (0.42–0.93)	0.021	1.16 (0.81–1.67)	0.412
Recipient age	0.99 (0.98–1.01)	0.448	1.01 (1.00–1.03)	0.107
Hepatitis C virus infection	0.67 (0.42–1.06)	0.089	1.37 (0.95–1.98)	0.088
Hepatitis B virus infection	0.79 (0.47–1.33)	0.375	1.43 (0.95–2.14)	0.083
Alcoholic liver disease	0.76 (0.44–1.30)	0.319	1.13 (0.74–1.73)	0.582
Primary sclerosing cholangitis	0.40 (0.16–1.01)	0.052	0.90 (0.50–1.64)	0.737
Primary biliary cirrhosis	0.42 (0.15–1.17)	0.097	0.85 (0.43–1.67)	0.633
Experience of the transplant team^a^	0.93 (0.89–0.98)	0.009	0.96 (0.90–1.01)	0.133
Intraoperative PRBC transfusions	1.15 (1.11–1.19)	<0.001	1.04 (1.01–1.08)	0.011
Intraoperative FFP transfusions	1.14 (1.10–1.17)	<0.001	1.03 (1.00–1.07)	0.088
Duration of cold ischemia	1.24 (1.10–1.40)	<0.001	1.10 (1.00–1.21)	0.062
Donor age	1.01 (0.99–1.03)	0.204	1.01 (1.00–1.03)	0.046
Multivariable analyses				
Hepatocellular cancer			1.88 (1.18–3.01)	0.008
MELD score	2.00 (1.57–2.54)	<0.001		
Male recipient sex	0.63 (0.35–1.10)	0.104		
Experience of the transplant team^a^	0.99 (0.91–1.07)	0.772		
Intraoperative PRBC transfusions	1.12 (1.02–1.24)	0.022	1.04 (1.01–1.08)	0.007
Intraoperative FFP transfusions	0.98 (0.89–1.08)	0.669		
Duration of cold ischemia	1.20 (1.04–1.39)	0.013		
Donor age			1.01 (1.00–1.03)	0.106

Odds ratios and hazard ratios were provided per: 10-point increase for MELD score; 1-year increase for recipient and donor age; 100 transplantations increase for experience of the transplant team; 1-unit increase for blood and plasma transfusions; and 1-hour increase for duration of cold ischemia

^a^Defined as a cumulative number of previously performed transplantations in the department

*OR* odds ratio, *HR* hazard ratio, *CI* confidence interval, *MELD* model for end-stage liver disease, *PRBC* packed red blood cells, *FFP* fresh frozen plasma

**Fig. 1:**
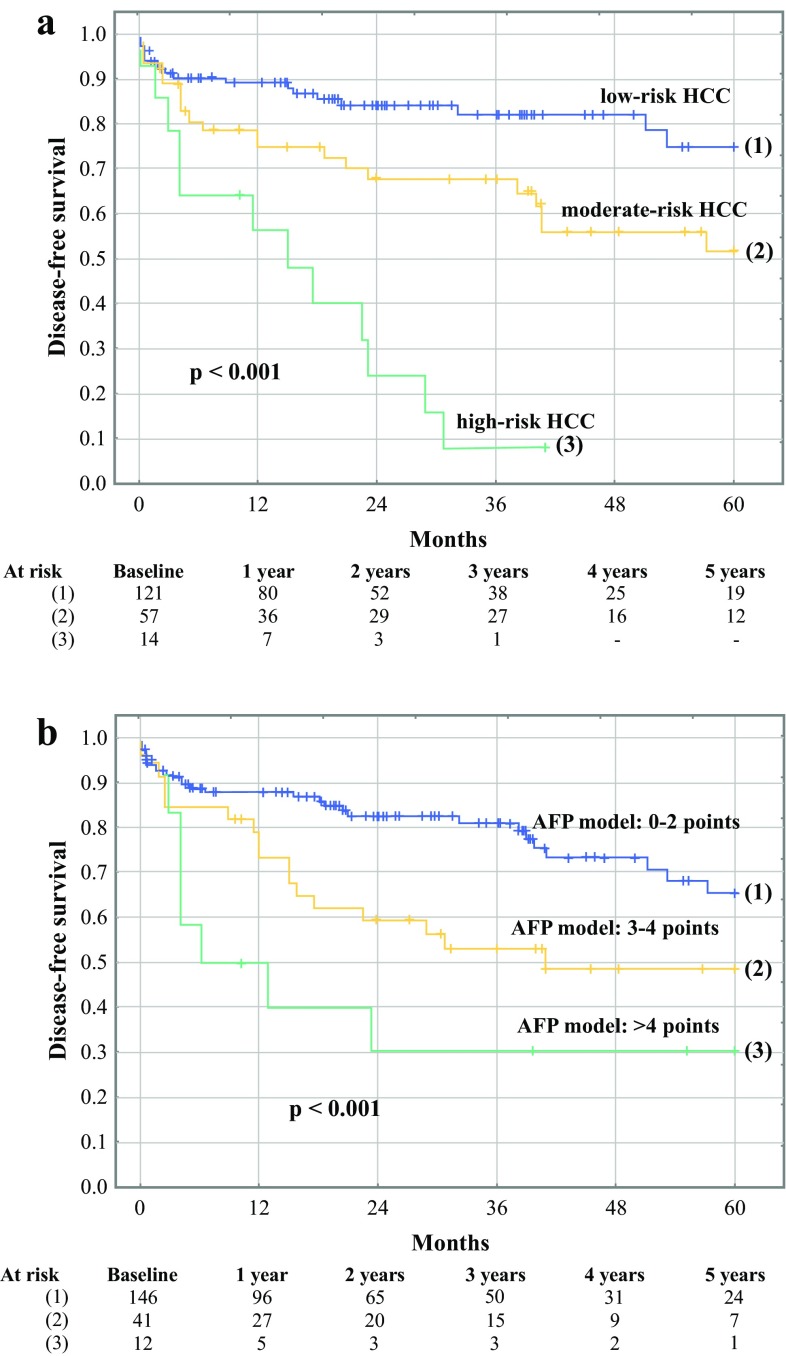
Disease-free survival of low-risk, moderate-risk, and high-risk hepatocellular cancer (HCC) patients (**a**) and those with AFP model of 0–2, 3–4, and >4 points (**b**) after liver transplantation. Numbers of patients at risk are presented at the *bottom*

Disease-free survival for all HCC patients at 1, 3, and 5 years was 82.1, 68.3, and 57.8%, respectively. Risk factors for worse 5-year disease-free survival on univariable analyses comprised pretransplant alpha-fetoprotein (*p* < 0.001), total tumor volume (*p* < 0.001), tumor size (*p* = 0.013), number (*p* = 0.006), and poor differentiation (*p* = 0.035; Table 3). Pretransplant alpha-fetoprotein (*p* = 0.013) and total tumor volume (*p* = 0.008) were the independent risk factors. The optimal cutoffs for predicting HCC recurrence were 175 ng/mL for alpha-fetoprotein and 65.4 cm^3^ for total tumor volume (Supplementary Fig. 2). The corresponding AUCs were 0.739 (95% CI 0.608–0.870) and 0.725 (95% CI 0.603–0.848), respectively. Bivariable analysis confirmed the independent impact of pretransplant alpha-fetoprotein (*p* < 0.001; HR 2.88, 95% CI 1.66–5.00) and total tumor volume (*p* = 0.002; HR 2.35, 95% CI 1.35–4.07) on disease-free survival following their transformation to categorical variables. Basing on the established cutoffs, HCC patients were assigned 1 point for pretransplant alpha-fetoprotein ≥175 ng/mL and 1 point for total tumor volume ≥65.4 cm^3^. The newly proposed risk score was associated with an AUC of 0.886 in prediction of recurrence (95% CI 0.816–0.956). Patients with 0, 1, and 2 points were categorized as low-risk, moderate-risk, and high-risk, respectively. The rates of low-risk, moderate-risk, and high-risk profiles in patients within the Milan criteria were 82.8% (*n* = 96), 15.5% (*n* = 18), and 1.7% (*n* = 2), respectively, compared with the corresponding rates of 32.9% (*n* = 25), 51.3% (*n* = 39), and 15.8% (*n* = 12), respectively, in patients beyond the Milan criteria (*p* < 0.001). Disease-free survival rate at 5 years was 74.9% for the low-risk HCC patients, 51.7% for the moderate-risk HCC patients, and 8.0% (3-year) for the high-risk HCC patients (*p* < 0.001; Fig. 1a). Application of the AFP model for prediction of recurrence was associated with an AUC of 0.845 (95% CI 0.778–0.913). Disease-free survival rates at 5 years were 65.5, 48.5, and 30.0% in patients with 0–2, 3–4, and >4 points in the AFP model, respectively (*p* < 0,001; Fig. 1b).

**Table 3:** Analyses of risk factors for worse 5-year disease-free survival after liver transplantation in patients with hepatocellular cancer

Factor	Univariable		Multivariable	
	HR (95% CI)	*P*	HR (95% CI)	*P*
Size of the largest tumor	1.17 (1.03–1.32)	0.013		
Number of tumors	1.13 (1.04–1.23)	0.006		
Total tumor volume	1.01 (1.00–1.01)	<0.001	1.05 (1.01–1.09)	0.008
Microvascular invasion	1.58 (0.93–2.67)	0.089		
Poor tumor differentiation	2.02 (1.05–3.90)	0.035		
Alpha-fetoprotein	1.20 (1.08–1.33)	<0.001	1.20 (1.04–1.38)	0.013
Neoadjuvant treatment	1.20 (0.72–2.01)	0.487		
Male recipient sex	0.71 (0.41–1.23)	0.223		
Recipient age	1.02 (0.99–1.05)	0.296		
MELD score	1.03 (0.99–1.07)	0.129	1.05 (0.99–1.11)	0.086
Hepatitis C virus infection	1.04 (0.61–1.77)	0.896		
Hepatitis B virus infection	1.12 (0.67–1.88)	0.656		
Alcoholic liver disease	0.94 (0.48–1.85)	0.855		
Intraoperative PRBC transfusions	1.03 (0.99–1.06)	0.104		
Intraoperative FFP transfusions	1.02 (0.97–1.06)	0.466		
Duration of cold ischemia	1.14 (0.99–1.32)	0.066		
Donor age	1.01 (0.99–1.03)	0.536		

Hazard ratios were provided per: 1-cm increase for tumor size; 1 increase for tumor number; 10 cm^3^ increase for total tumor volume; 1 natural logarithm increase for alpha-fetoprotein; 1-year increase for recipient and donor age; 1-point increase for MELD; 1 unit increase for transfusions; and 1-hour increase for duration of cold ischemia

*HR* hazard ratio, *CI* confidence interval, *MELD* model for end-stage liver disease, *PRBC* packed red blood cells, *FFP* fresh frozen plasma

**Fig. 2:**
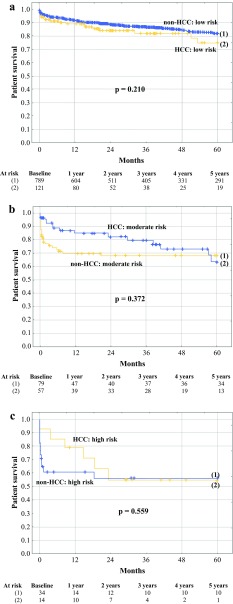
Survival of low-risk (**a**), moderate-risk (**b**), and high-risk (**c**) hepatocellular cancer (HCC) patients and non-HCC patients after liver transplantation. Numbers of patients at risk are presented at the *bottom*

Basing on MELD score, non-HCC patients were divided into low-risk, moderate-risk, and high-risk subgroups including patients with MELD <30, 30–40, and >40, respectively. Patient survival differed significantly between the three non-HCC subgroups (*p* < 0.001). Low-risk HCC patients exhibited 5-year survival rate of 74.9%, similar to that observed for the entire cohort of non-HCC patients (78.5%; *p* = 0.932). Conversely, 5-year survival rate for moderate-risk HCC patients (63.3%) was nonsignificantly (*p* = 0.191), yet remarkably, lower than that observed for non-HCC patients in general, whereas the corresponding survival of high-risk HCC patients (55.0%) was significantly compromised (*p* = 0.019). However, there were no significant differences with respect to 5-year survival between low-risk HCC patients and low-risk non-HCC patients (74.9% vs. 81.9%, respectively; *p* = 0.210; Fig. 2a), between moderate risk HCC patients and moderate-risk non-HCC patients (63.3% vs. 68.0%, respectively; *p* = 0.372; Fig. 2b), and between high-risk HCC patients and high-risk non-HCC patients (55.0% vs. 56.0%, respectively; *p* = 0.559; Fig. 2c). Patients with HCC and AFP model of 0–2 points tended to exhibit lower 5-year survival rate (68.0%) than low-risk non-HCC patients (*p* = 0.082), whereas survival of HCC patients with AFP model of 3–4 points (69.9%) and >4 points (50.0%) was similar to that observed for moderate-risk (*p* = 0.469) and high-risk (*p* = 0.521) non-HCC patients, respectively (Supplementary Fig. 3). The rates of HCC patients classified as low-, moderate-, and high-risk of negative outcomes were 63.0% (*n* = 121), 29.7% (*n* = 57), and 7.3% (*n* = 14), respectively, compared with the corresponding rates of 87.5% (*n* = 789), 8.8% (*n* = 79), and 3.8% (*n* = 34), respectively, in non-HCC patients (*p* < 0.001).

## Discussion

LT for HCC was historically associated with extremely poor long-term outcomes, characterized by a median survival of approximately 1.5 years and 5-year survival rate of approximately 20%.^22^ Limitations in patient selection defined by the Milan criteria led to exclusion of high-risk patients and thus to a remarkable improvement of outcomes.^4^ These criteria remained the benchmark for selection of HCC patients for LT to keep the low risk of tumor recurrence and provide survival rates at 5 years comparable to non-HCC patients.^23^ Since their introduction, HCC patients within and beyond the Milan criteria started to be considered as two distinct populations, as opposed to non-HCC patients despite the heterogeneity of posttransplant outcomes of the latter. The results of the present study oppose this utility-based barrier for selection of moderate and high-risk HCC patients, because it is not used for exclusion of moderate- and high-risk non-HCC patients exhibiting similar survival outcomes.

While division of HCC patients into subgroups was based on total tumor volume and alpha-fetoprotein, the two well-known predictors of HCC recurrence, division of non-HCC patients was based solely on laboratory MELD score, commonly used for prioritization purposes and also known to influence negatively posttransplant outcomes.^8^,^9^,^11^,^12^,^24^ The presented survival outcomes of high-risk HCC patients are even lower than that reported in the literature for patients beyond the Milan criteria, presumably due to more liberal selection policy applied in the authors department reducing selection bias.^5^,^7^,^12^,^25^,^26^ The outcomes of high-MELD non-HCC patients seem comparable to that previously reported.^24^ Although survival of HCC patients in general and, in particular, that of high-risk HCC patients was significantly inferior to that observed for the entire cohort of non-HCC patients, the outcomes were similar in HCC and non-HCC patients belonging to low-, moderate-, and high-risk subgroups. Moreover, patients with lower score in the AFP model tended to have survival outcomes lower than the low-risk non-HCC recipients. On the contrary, survival of HCC patients with moderate and high number of points in the AFP model highly resembled that of moderate-risk and high-risk non-HCC patients. Therefore, the utility principle appears to be unequally applied in limiting access to transplantation for HCC patients. Notably, 5-year survival in none of the studied subgroups was below the threshold of 50%. The discrepancy between the >50% 5-year survival observed for high-risk HCC patients in the present study and the 20% historical rate is potentially related to an overall improvement of transplantation results, an argument currently raised in the discussion on reinitiation of transplantation for unresectable colorectal cancer metastases.^21^,^24^,^27^–^29^

Apart from HCC and high MELD scores, several other conditions are reported to be associated with particularly poor posttransplant outcomes resembling that of the highest-risk HCC patients, such as retransplantation for hepatitis C virus recurrence, trauma, and unresectable neuroendocrine tumors metastases.^25^,^30^,^31^ Considering survival benefit, it is the high-risk HCC patients and non-HCC patients that are reported to benefit most from undergoing LT.^18^,^32^,^33^ However, pretransplant alpha-fetoprotein was previously found to be inversely correlated with transplant benefit.^34^ Therefore, potential expansion of the selection criteria into patients with high alpha-fetoprotein should be considered with great caution considering both transplant utility and benefit. Nevertheless, high-risk cirrhotic HCC patients with high alpha-fetoprotein and concomitant excessive tumor burden are unlikely candidates for effective nontransplant therapies, particularly liver resection, which potentially increases transplant benefit. Notably, initiating aggressive therapeutic strategies was recently found to approximately double median survival compared with the use of targeted therapy, chemotherapy, or best supportive care even in HCC patients with compromised performance status.^35^

Irrespective of survival outcomes comparable to non-HCC patients in the corresponding risk groups, selection of highest-risk HCC patients for LT with expected 3-year disease-free survival below 10% would be extremely controversial. However, a major proportion of patients with post-transplant HCC recurrence is amenable to effective treatment and thus, recurrences within 5 posttransplant years should not necessarily be considered as failures when considering survival outcomes at this timeframe.^36^ Remarkable differences between 5-year patient and disease-free survival also were reported previously.^12^ From the survival perspective, there is currently no reason to consider HCC recurrence differently from posttransplant recurrences of benign conditions, such as excessive alcohol consumption or cholestatic diseases, because the latter also are associated with allograft failure and long-term mortality.^37^,^38^

The most controversial issue related to broadening access to transplantation for HCC patients is its impact on wait-list dynamics for non-HCC patients. Even with the current selection strategies, HCC patients are unjustifiably privileged under the MELD-based allocation system with HCC exceptions.^15^ Several solutions for reestablishing equity in organ allocation were recently proposed.^16^,^34^,^39^ Their introduction into clinical practice may partially ameliorate the negative impact of increasing number of HCC candidates. However, these proposals do not have the capacity to solve the inferior position of HCC patients beyond the current criteria, who are excluded from transplant therapy and exhibit extremely poor outcomes. In contrast to a median overall survival of approximately 5 years for HCC patients in the high-risk subgroup found in the present study, median overall survival of only 13.6 months for Child A patients and only 5.2 months for Child B patients receiving targeted therapy was recently reported by the authors of the GIDEON study.^40^

Besides those inherent to its retrospective design, the present study has several limitations. First, the score for division of HCC patients into the low-, moderate-, and high-risk group was not validated and may not be accurate for other HCC populations. However, it was not created for further use or implementation in selection of patients, but for division of the HCC cohort to subgroups with different risk profiles only for the purposes of this study. Importantly, the results remained similar following division of HCC patients according to the previously proposed AFP model.^6^ Moreover, MELD was used for corresponding division of non-HCC patients instead of using a more complicated score in order to demonstrate that 1 single variable, already used to define urgency for allocation purposes, can be implemented to categorize patients on utility basis. Furthermore, survival of HCC was nonsignificantly lower than non-HCC patients in low-, moderate-, and high-risk groups, posing a risk of type II error. However, the differences do not seem clinically significant as they ranged from 1 to 7% at 5 years. Finally, the outcomes for particular risk groups among HCC patients may be overestimated due to selection bias. However, the reported survival outcomes are supported by the wide range of disease-free survival rates observed for particular HCC subgroups, with 3-year rate below 10% being expected for the highest-risk patients.

In conclusion, the presented results provide rationale for the discussion on reinitiation of LT for patients with more advanced HCC stages. The current low-risk selection policy for HCC patients appears to be an unjust barrier to LT, not applied for high-risk non-HCC patients.

## Electronic supplementary material

Below is the link to the electronic supplementary material.

Supplementary Fig. 1 Overall survival of hepatocellular cancer (HCC) patients and non-HCC patients after liver transplantation. Numbers of patients at risk are presented at the bottom. Supplementary material 1 (TIFF 573 kb)

Supplementary Fig. 2 Receiver operating characteristics curves for pre-transplant alpha-fetoprotein concentration (**a**) and total tumor volume (**b**) in prediction of hepatocellular cancer recurrence after liver transplantation. Areas under curves (AUCs) are presented with 95% confidence intervals (95% CIs). Supplementary material 2 (TIFF 1434 kb)

Supplementary Fig. 3 Survival of hepatocellular cancer (HCC) patients with AFP model of 2 (**a**), 3–4 (**b**), and >4 (**c**) and non-HCC patients belonging to low-risk, moderate-risk, and high-risk groups, respectively, after liver transplantation. Numbers of patients at risk are presented at the bottom. Supplementary material 3 (TIFF 1720 kb)
