# Supplementary figures and images for: Challenging the principle of utility as a barrier for wider use of liver transplantation for hepatocellular cancer
Source: Ann Surg Oncol. 2017 Jul 10;24(11):3188–95. doi: 10.1245/s10434-017-5989-x (PMC5596049; doi:10.1245/s10434-017-5989-x)

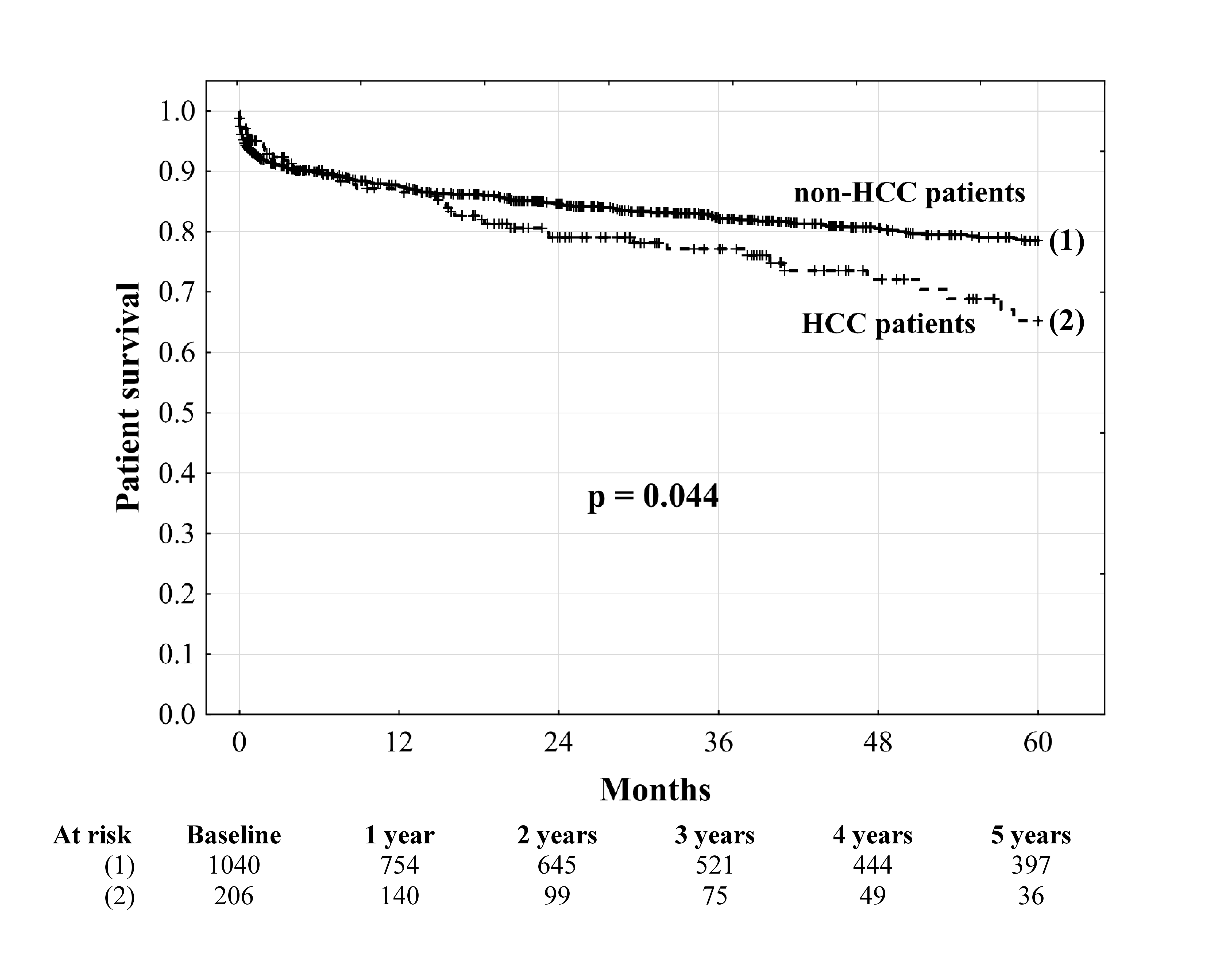

Supplement: Supplementary file 1 — Supplementary Fig. 1 Overall survival of hepatocellular cancer (HCC) patients and non-HCC patients after liver transplantation. Numbers of patients at risk are presented at the bottom. Supplementary material 1 (TIFF 573 kb) [file 10434_2017_5989_MOESM1_ESM.tif]

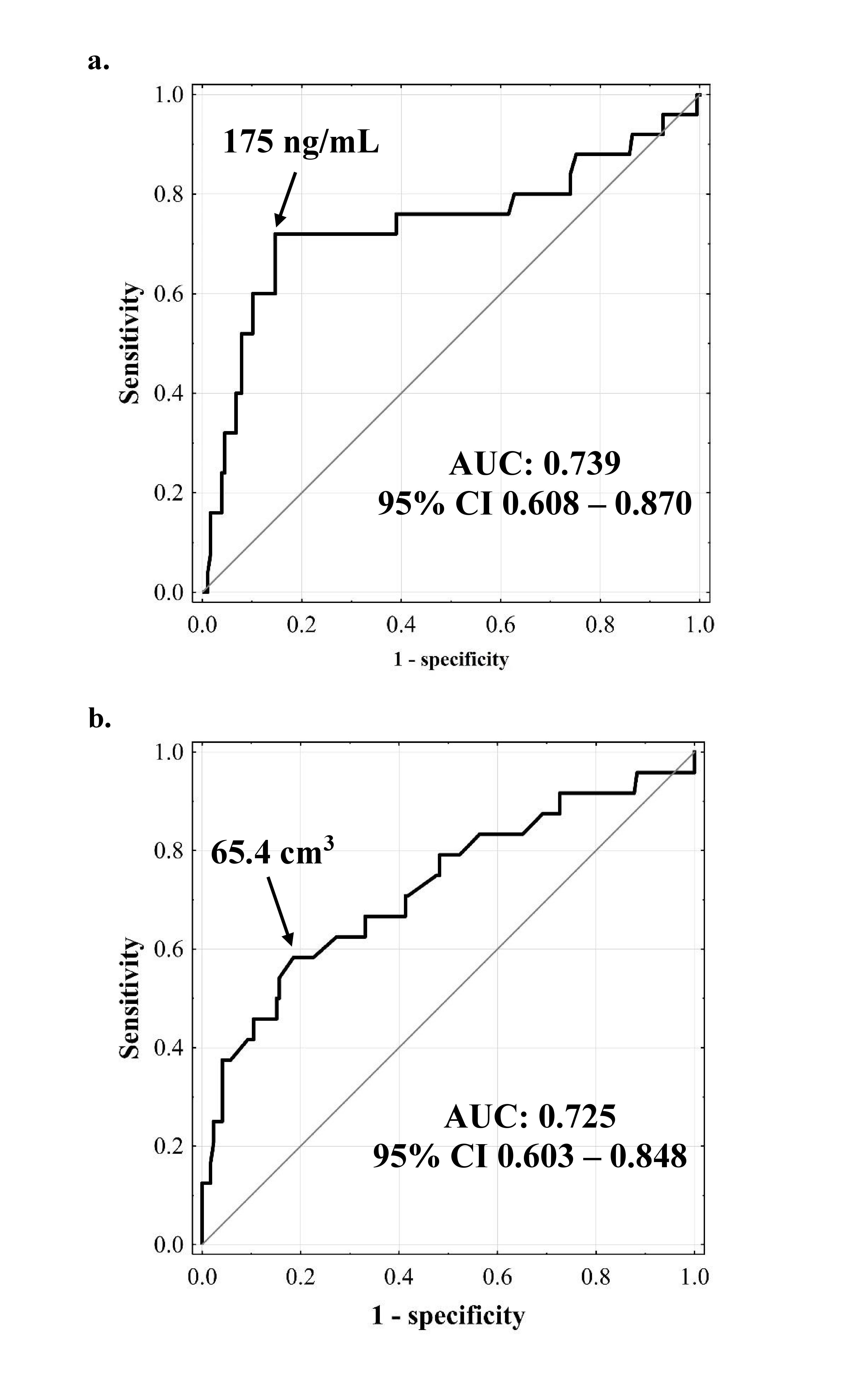

Supplement: Supplementary file 2 — Supplementary Fig. 2 Receiver operating characteristics curves for pre-transplant alpha-fetoprotein concentration (a) and total tumor volume (b) in prediction of hepatocellular cancer recurrence after liver transplantation. Areas under curves (AUCs) are presented with 95% confidence intervals (95% CIs). Supplementary material 2 (TIFF 1434 kb) [file 10434_2017_5989_MOESM2_ESM.tif]

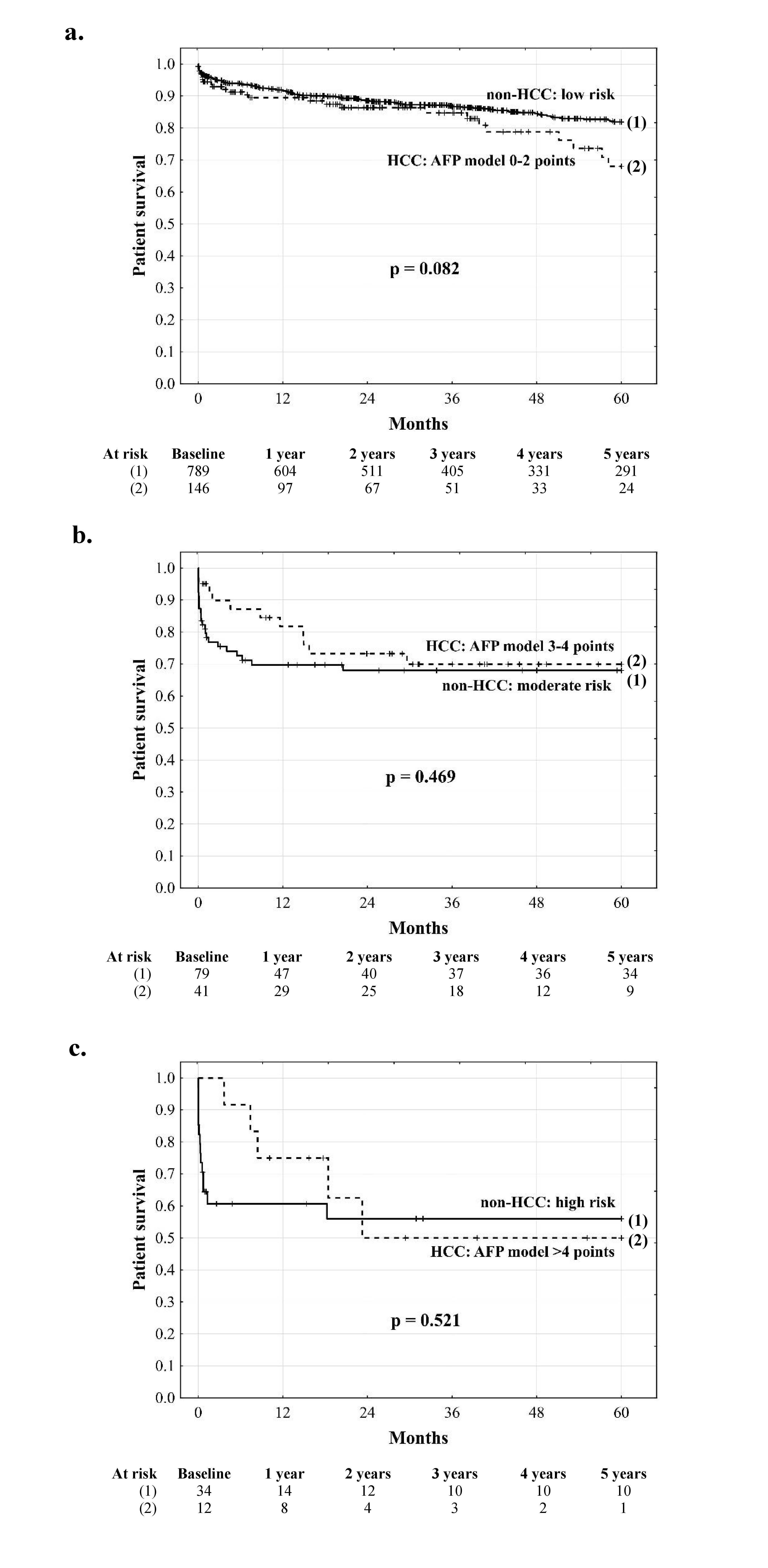

Supplement: Supplementary file 3 — Supplementary Fig. 3 Survival of hepatocellular cancer (HCC) patients with AFP model of 2 (a), 3–4 (b), and >4 (c) and non-HCC patients belonging to low-risk, moderate-risk, and high-risk groups, respectively, after liver transplantation. Numbers of patients at risk are presented at the bottom. Supplementary material 3 (TIFF 1720 kb) [file 10434_2017_5989_MOESM3_ESM.tif]
